# Anti‐obesity effect of *Melandrium firmum* Rohrbach extract in 3T3‐L1 cells and high‐fat diet‐induced obese C57BL/6N mice

**DOI:** 10.1002/fsn3.1466

**Published:** 2020-04-08

**Authors:** Hyun‐Yong Kim, Ju Hee Kim, Guanglei Zuo, Soon Sung Lim

**Affiliations:** ^1^ Department of Food Science and Nutrition Hallym University Chuncheon Korea; ^2^ Institute of Natural Medicine Hallym University Chuncheon Korea; ^3^ Institute of Korean Nutrition Hallym University Chuncheon Korea

**Keywords:** adipogenesis, anti‐obesity, lipid profile, lipogenesis, *Melandrium firmum* Rohrbach, RT‐PCR

## Abstract

In this study, we first investigated the influence of *Melandrium firmum* Rohrbach (MF) on the accumulation of lipid content in 3T3‐L1 cells and in vitro results showed that MF extraction suppressed the differentiation of 3T3‐L1 pre‐adipocytes in a concentration‐dependent manner without showing cytotoxicity. Hence, we studied the effects of MF on preventing obesity in C57BL/6N mice. The results showed that MF decreased food efficiency ratio, body weight, epididymal adipose and hepatic tissue weight, hepatic lipid metabolites, and triacylglycerol and cholesterol serum levels, when compared with the high‐fat diet group. Moreover, MF significantly inhibited the expression of genes related to adipogenesis, such as PPAR‐γ, C/EBP‐α, and aP2, and those related to lipogenesis, such as SREBP‐1c, FAS, SCD‐1, and CD36 in epididymal adipose and liver tissues. These anti‐adipogenic and anti‐lipogenic effects of MF suggest that it could be used as a food including potential functional ingredient to prevent high‐fat diet‐induced obesity.

## Graphical abstract

We investigated the influence of Melandrium firmum Rohrbach on the accumulation of lipid content in 3T3‐L1 cells, and in vitro results showed that MF extraction suppressed the differentiation of 3T3‐L1 pre‐adipocytes in a concentration‐dependent manner without showing cytotoxicity. Hence, we studied the effects of MF on preventing obesity in C57BL/6N mice. Anti‐adipogenic and antilipogenic effects of MF suggest that it could be used as a functional food to prevent high‐fat diet‐induced obesity.

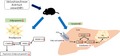

## 1 INTRODUCTION

In the industrialized world, obesity is one of the epidemics associated with an increased risk of other life‐threatening pathologies (Ravinet Trillou et al., 2003). Obesity results from the disequilibrium in energy intake and expenditure, and is known to be a risk factor for type 2 diabetes (Ono, Hattori, Fukaya, Imai, & Ohizumi, 2006). Obesity is caused by the generation of new adipocytes (You, Lee, Kim, Kim, & Chang, 2014), and adipogenesis is a process of cell differentiation where pre‐adipocytes mature into adipocytes (Wang, Hwang, Kim, & Lim, 2017). Adipogenesis is a complex process that is accompanied by changes in morphology, hormones, and gene expression. CCAAT/enhancer‐binding protein (C/EBP) transcription factor family and peroxidase proliferator‐activated receptor γ (PPAR γ) act to regulate adipocyte differentiation (Lee et al., 2010), sterol regulatory element‐binding protein (SREBP‐1c) acts as a stimulator (Ali, Hochfeld, Myburgh, & Pepper, 2013), and adipocyte P2(aP2) and fatty acid synthase (FAS) also play a role in the maturation of adipocytes (Choi et al., 2000). Lipogenesis involves fatty acid and triglyceride synthesis in the liver, and fatty acid synthase (FAS), acetyl‐CoA carboxylase (ACC), and fatty acid esterification transcriptional factors like stearoyl‐CoA desaturase (SCD‐1) play an important role in lipogenesis in the adipose and liver tissues (Wang et al., 2017).

*Melandrium firmum* Rohrbach (MF) is a widely distributed plant in Korea (Zheng et al., 2008). The dried aerial part of MF has been used as a medicine for acute nephritis and liver cirrhosis in China, and to treat breast cancer and lactation disorders in Korea (Zhang et al., 2015). MF contains several classes of compounds like sapogenin, saponin, flavonoids, and triterpenoids, and their pharmacological effects have been evaluated (Lee et al., 2012). The root extract of MF showed apoptotic effects in SHSY5Y neuroblastoma cells, and butanol fraction of the methanol extraction of whole body of MF exhibited anti‐inflammatory activity. The methanol extract of MF inhibited the development of benign prostatic hyperplasia (BPH) in rats (Chandra & Rawat, 2015).

In this study, we examined the anti‐adipogenic effects of MF extract using Oil Red O staining in 3T3‐L1 adipocytes. In addition, the anti‐obesity effects of the extract were investigated by measuring body weight gain, serum and lipid profiles, histological variation in the adipose tissue, and expression of the genes associated with adipogenesis and lipogenesis for 10 weeks in high‐fat diet‐fed obese mice.

## 2 MATERIALS AND METHODS

### 2.1 Plant material and preparation of the extract

The whole plant of MF was purchased from Jeongjin Distribution Co, Korea. The dried MF (1.5 kg) was pulverized, and the extract was obtained using 70% ethanol (15 L) at 25℃ for 48 hr. The MF extracts were filtered using filter paper (Hyundai Micro No. 20) and concentrated by a reduced pressure evaporator (N‐1000; Tokyo Rikakikai) to obtain the extract powder.

### 2.2 3T3‐L1 cell culture and treatment

3T3‐L1 murine pre‐adipocytes were obtained from American Type Culture Collection and cultured to confluence at 37°C under a humidified 5% CO_2_ atmosphere in Dulbecco's modified Eagle's medium (DMEM, Gibco), including 10% bovine calf serum (GenDEPOT) and 100 U/ml penicillin–streptomycin (Gibco). Two days after the cells had reached confluence (day 0), pre‐adipocytes of 3T3‐L1 were cultured in differentiation medium (DM) containing 10% fetal bovine serum (FBS; Gibco), 10 μg/ml insulin (Sigma‐Aldrich), 0.5 mM 3‐isobutyl‐1‐methylxanthine (IBMX, Sigma‐Aldrich), and 1 μM dexamethasone (Sigma‐Aldrich). Two days after stimulation with differentiation inducer (MDI (methylisobutylxanthine), including 0.5 mM IBMX, 1 μM dexamethasone, and 10 μg/ml insulin) (day 2), the medium was changed to a 10% FBS/DMEM medium containing 10 μg/ml insulin. After another two days (day 4), the medium was changed to 10% FBS/DMEM medium and cultured in 10% FBS/DMEM medium every 2 days. Full differentiation was achieved by day 8. During differentiation, the MF extracts were used, at concentrations of 10 and 50 μg/ml, to inhibit the differentiation of adipocytes in the 3T3‐L1 culture between days 0 and 4.

### 2.3 Oil Red O staining and determination of lipid content

Cells were stained with Oil Red O solution (Sigma‐Aldrich), to investigate both adipogenic potential and lipid accumulation. On day 8, the cultured 3T3‐L1 cells were washed with cold phosphate‐buffered saline (PBS) and then fixed with 10% formaldehyde at 25℃. The cells were stained with filtered 0.5 μg/ml Oil Red O solution (0.5 g of Oil Red O in 500 ml of isopropyl alcohol) and washed twice. The lipid droplets were dissolved in isopropanol, and absorbance was measured at 540 nm using a microplate reader (Sensident scan, Labsystems).

### 2.4 Cell viability assay

3‐(4,5‐dimethylthiazol‐2‐yl)‐5‐(3‐carboxymethoxyphenyl)‐2‐(4‐sulfophenyl)‐2H‐tetrazolium, inner salt (MTS) assay kit (Promega) was used according to the manufacturer's instructions to investigate cell viability of 3T3‐L1 cells upon MF extract treatment. 3T3‐L1 cells (5 × 10^3^/well) were cultured in 96‐well plates and treated with MF extract (10 and 50 μg/ml). The optical density was measured at 490 nm thrice using a microplate reader (Sensident scan).

### 2.5 Animals and their diet

All animal experiments were conducted after obtaining approval of the Institutional Animal Care and Use Committees (IACUC) of Hallym University (Hallym‐2015–12‐R). Male C57BL/6N mice (5‐week old) were purchased from Central Lab Animal (SLC). After a week's rest, mice were randomly allocated to one of the four diet groups (*n* = 6 per group), normal‐fat diet (NFD), high‐fat diet (HFD), HFD supplemented with 1% (10 g/kg) *Garcinia cambogia* extract (GR; ESFood), and HFD supplemented with 1% (10 g/kg) MF. *Garcinia cambogia* extract containing 60% (‐)‐hydroxycitric acid was used as a positive control because of its anti‐adipogenic and anti‐lipogenic activities (Kwon et al., 2003) (Kang et al., 2013) (Rodgers, Tschop, & Wilding, 2012). The experimental diets were based on the AIN‐93 diet, and the HFD contained 60% fat (lard, 310 g/kg and soybean oil, 30 g/kg). *Garcinia cambogia* extract and MF were dissolved in corn oil and added to the experimental diet. The diets of two groups (GR, MF) were prepared by DooYeol Biotech (Seoul, Korea), and their compositions are shown in Table 1. Mice were housed under controlled temperature and lighting (22 ± 2°C and 50 ± 10% humidity with a 12‐hr light and dark cycle) with free access to water and food. Mice followed the experimental diet for 10 weeks. Body weight was measured twice per week, and food intake was recorded daily.

**Table 1:** Compositions of experimental diets (in g/kg)

Group^1^	NFD	HFD	GR	MF
Casein	210	265	265	265
L‐cystine	3	4	4	4
Corn starch	280	–	–	–
Maltodextrin	50	160	150	150
Sucrose	325	90	90	90
Lard	20	310	310	310
Soybean oil	20	30	30	30
Cellulose	37.15	65.5	65.5	65.5
Mineral mixure^2^	35	48	48	48
Vitamin mix^3^	15	21	21	21
Calcium phosphate, dibasic	2	3.4	3.4	3.4
Choline bitartrate	2.75	3	3	3
Yellow food color	0.1	–	–	–
Blue food color	–	0.1	0.1	0.1
*Garcinia cambogia* extract of 60% (−)‐hydroxycitric acid	–	–	10	–
*Melandrium firmum* Rohrbach extract	–	–	–	10
Total	1,000	1,000	1,000	1,000

^1^ NFD, normal‐fat diet; HFD, high‐fat diet; GR, HFD + 1% *Garcinia cambogia* extract; MF, HFD + 1% *Melandrium firmum* Rohrback extract.

^2^ Mineral mixture according to AIN‐93G‐MX (94046).

^3^ Vitamin mixturVe according to AIN‐93‐VX (94047).

### 2.6 Collection of serum and tissue samples

After 10 weeks, all mice were sacrificed following a 12‐hr fasting, and tissues were collected for analysis. Blood was collected from the inferior vena cava and was subjected to centrifugation at 2,090 *g* at 4°C for 15 min to separate the serum. The epididymal adipose tissue and liver were removed, weighed, and stored at 80°C until analysis.

### 2.7 Biochemical analysis

Levels of triacylglycerol (TG), high‐density lipoprotein (HDL) cholesterol, low‐density lipoprotein (LDL) cholesterol, serum alanine aminotransferase (ALT), aspartate aminotransferase (AST), blood urea nitrogen (BUN), and creatinine (CREA) in serum were measured with commercial kits (981,786, 981,823, 981,656, 981,769, 981,771, 981,820, and 981,811, respectively, Thermo Electron Corporation, Vantaa, Finland) using Thermo Fisher Konelab 20XTi Analyzer (Thermo Electron Corporation, SeoKwang LABOTECH).

### 2.8 Histological analysis

Epididymal adipose tissue was fixed with 4% formaldehyde and embedded in paraffin. Sections (5 μm thick) were cut, and each section was stained with hematoxylin and eosin (H & E). All the sections were photographed using an optical microscope (Leica RM2235, Wetzlar, Germany) and printed at a final magnification of 200×. Images were observed using a microscope (Axiomager), and the diameter of each adipocyte was analyzed using the AxioVisionRel. 4.8 software (Zeiss).

### 2.9 RNA extraction, cDNA synthesis, and real‐time PCR

Total RNA was extracted from the epididymal adipose tissue using an Easy‐Blue kit (Intron Biotechnology Inc) according to the protocol provided by the manufacturer. Then, total RNA was quantified with a NanoDrop‐2000 (Thermo Fisher Scientific). cDNA was synthesized (an equal amount of total RNA) with the Moloney murine leukemia virus transcriptase and Oligo (dT) 15 primers (Promega) using a Life Touch thermal cycler (Life Eco, Bioer Technology). The program was set for 1 hr of initiation at 42°C, followed by 10 min of incubation at 95°C and 10 min at 4°C. RT‐PCR was performed using the QuantiTect SYBR Green PCR kit (Qiagen), according to the manufacturer's instructions. cDNA was amplified for 40 cycles of denaturation (95°C for 30 s), annealing (57°C for 40 s), and extension (72°C for 40 s) using a RotorGene RG3000 real‐time PCR machine (Corbett Research). The purity of the PCR product was determined using melting curve analysis. The relative quantification of the expression of each gene was calculated using the comparative threshold cycle (Ct) method (Applied Biosystems). mRNA levels were normalized to β‐actin. Primer sequences are shown in Table 2.

**Table 2:** Sequence of primers used in real‐time PCR

Gene	Primer sequence(5'→3')	
	Forward primer	Reverse primer
β‐Actin	GTCGTACCACTGGCATTGTG	GCCATCTCCTGCTCAAAGTC
C/EBP‐α	AGACATCAAGCGCCTACATCG	TGTAGGTGCATGGTGGTCTG
PPAR‐γ	CCCTGGCAAACGATTTGTAT	AATCCTTGGCCCTCTGAGAT
SREBP−1c	GCGCTACCGGTCTTCTATCA	TGCTGCCAAAAGACAAGGG
CD36	TCCTCTGACATTTGCAGGTCTATC	GTGAATCCAGTTATGGGTTCCAC
SCD−1	CGAGGGTTGGTTGTTGATCTGT	ATAGCACTGTTGGCCCTGGA
FAS	GATCCTGGAACGAGAACAC	AGACTGTGGAACACGGTGGT
aP2	AACACCGAGATTTCCTTCAA	TCACGCCTTTCATAACACAT

### 2.10 NMR‐based hepatic metabolomics

NMR‐based hepatic metabolomic profiling analysis was performed according to a previous report with minor modifications (Zira et al., 2013). The lipophilic extracts, containing the lipid constituents of the liver, were used for ^1^HNMR spectroscopy. The liver tissue (0.1 g) was homogenized first in 1 ml chloroform/methanol (CHCl_3_/MeOH, 3:1, v/v) and then centrifuged at 22,500 *g* for 10 min at 4°C. The supernatant was collected and dried under a stream of nitrogen. The lipophilic extracts were reconstituted in 665 μl of deuterated chloroform/methanol (CDCl_3_/CD_3_OD, 3:1, v/v) including tetramethylsilane (TMS) as an internal standard in NMR analysis. ^1^H NMR spectra were recorded using a Bruker AV 400 instrument.

### 2.11 Statistical analysis

Data from individual experiments are expressed as a mean value ± standard error (SE). Comparisons were carried out using a Student's unpaired *t* test and a one‐way analysis of variance (ANOVA), as deemed appropriate. *p* < .05 was considered statistically significant.

## 3 RESULTS

### 3.1 Effect of MF extract on cell viability of pre‐adipocytes

**Figure 1:**
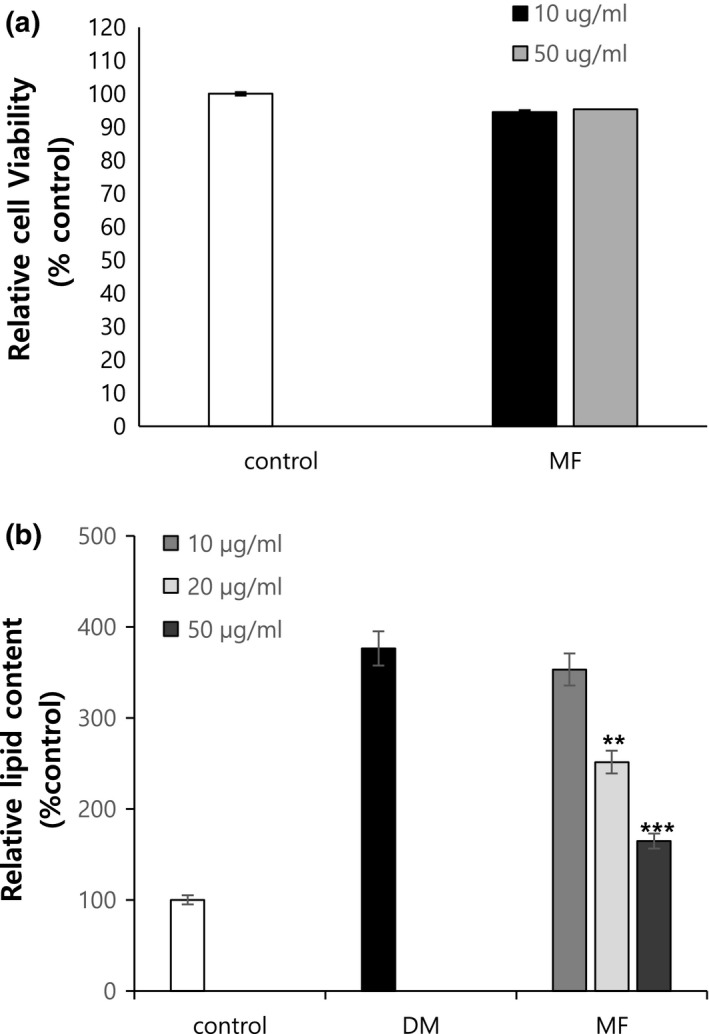
Effects of *Melandrium firmum* Rohrback extract on inhibition of 3T3‐L1 adipocyte differentiation and pre‐adipocyte viability. (a) 3T3‐L1 pre‐adipocytes were incubated with MF at different concentrations (10 and 50 µg/ml) for 24 hr. (b) Confluent 3T3‐L1 pre‐adipocytes were differentiated into adipocytes in medium with or without different concentration (10, 20, and 50 µg/ml) of MF extracts for 8 days. The results showed a significantly different compared to DM (**, *p* < .01; ***, *p* < .001). DM, differentiation media cells; MF, *Melandrium firmum* Rohrback extract

To determine the cell viability, an MTS assay was performed by treating 3T3‐L1 cells with MF extract (10 and 50 μg/ml). As shown in Figure 1a, MF showed no significant adverse effect on viability after 24 hr, indicating a noncytotoxic effect of MF on 3T3‐L1 cells.

### 3.2 Inhibitory effect of MF extract on lipid accumulation in 3T3‐L1 cells

The anti‐adipogenic effect of MF extract was evaluated using Oil Red O staining in differentiated 3T3‐L1 cells at concentrations of 10, 20, and 50 μg/ml. As shown in Figure 1b, treatment with MF extract significantly diminished relative lipid content in differentiated 3T3‐L1 adipocytes. Among the three concentrations, 50 μg/ml showed the highest inhibitory effect on adipogenesis.

### 3.3 Changes in body weight, food intake, and food efficiency ratio (FER)

**Figure 2:**
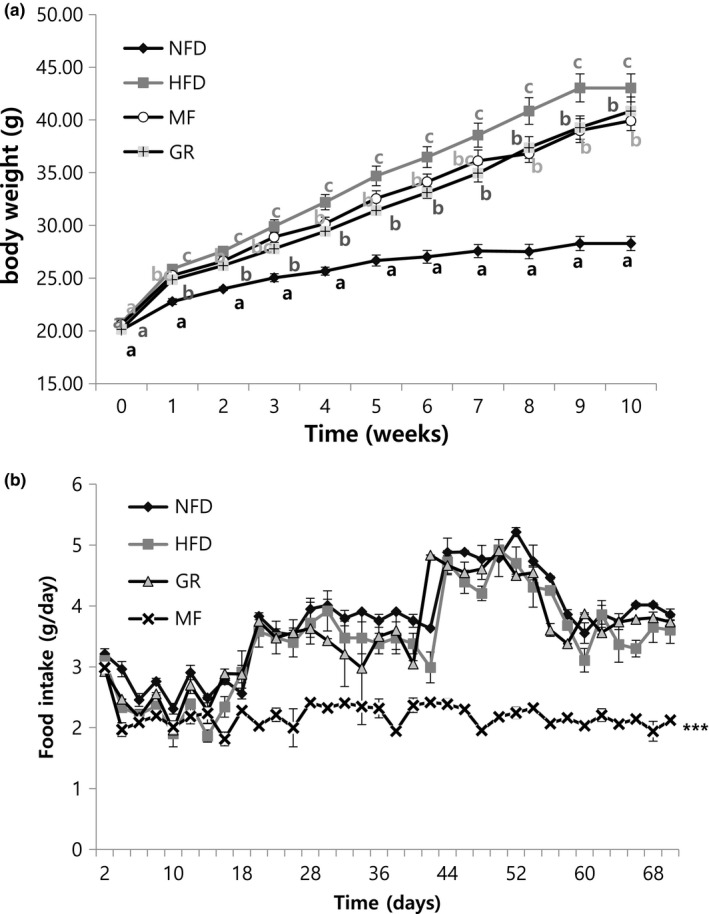
Effects of *Melandrium firmum* Rohrback extract on (a) body weight and (b) food intake in high‐fat diet‐induced obese mice. Results are presented as mean ± SE (*n* = 6). NFD, normal‐fat diet control; HFD, high‐fat diet control; GR, HFD + 1% *Garcinia cambogia* extract; MF, HFD + 1% *Melandrium firmum* Rohrback

In previous experiments, MF extract showed noncytotoxic and anti‐adipogenic effects on 3T3‐L1 cells. Hence, we decided to use MF extract for further in vivo anti‐obesity studies. The compositions of experimental diets are listed in Table 1. Figure 2a shows the difference in body weight. There was no significant difference during the early weeks, but after 10 weeks on the experimental diet, the body weight of HFD mice increased by 2.09‐fold than that of NFD mice. MF‐supplemented group (at 10 weeks) showed a significant decrease in body weight, by 1.08‐fold relative to the HFD group. Food intake showed no significant difference initially; however, after 3 weeks, there was a change in food intake in the MF‐supplemented group (Figure 2b). As shown in Table 3, body weight gain was affected by MF supplementation and was associated with a significant decrease in food intake. However, the food efficiency ratio (FER) of the MF group showed only a 1.24‐fold increase compared the HFD group during the 10‐week feeding period.

**Table 3:** Effects of *Melandrium firmum* Rohrback extract on body weight in high‐fat diet‐fed mice

Groups^1^	NFD	HFD	GR	MF
Body weight gain(g/10 weeks)	8.29 ± 0.74^2^ ^a^	24.05 ± 1.28^c^	19.27 ± 1.28^b^	19.24 ± 1.3^b^
Food intake(g/day)	3.74 ± 0.13^b^	3.41 ± 0.13^b^	3.51 ± 0.13^b^	2.2 ± 0.04^a^
FER^3^	2.22 ± 0.2^a^	7.06 ± 5.49^c^	5.49 ± 0.36^b^	8.74 ± 0.6^d^
Epididymal white adipose tissue weight (g)	1 ± 0.16^a^	2.31 ± 0.16^b^	2.18 ± 0.16^b^	2.31 ± 0.06^b^
Liver weight (g)	1.27 ± 0.19^a^	1.65 ± 0.38^a^	1.19 ± 0.07^a^	1.21 ± 0.13^a^

^1^ NFD, normal‐fat diet; HFD, high‐fat diet; GR, HFD + 1% *Garcinia cambogia* extract; MF, HFD + 1% *Melandrium firmum* Rohrback.

^2^ Results are presented as means ± SE (*n* = 6); values indicated with different letters are significantly different from each other at *p* < .05.

^3^ Food efficiency ratio (FER) = Body weight gain (g/day)/ Food intake (g/day).

### 3.4 Serum lipid profile and potential toxicity assessment

Table 4 shows the effects of HFD and MF administration on serum lipid profile. HFD group showed significantly increased serum TG and HDL cholesterol levels relative to the NFD group. When compared with the HFD group, there was a significant decrease in TG levels (1.43‐fold) and total cholesterol (2.04‐fold) in the MF group. HDL cholesterol levels were lower in the MF group relative to the HFD group, but HTR (HDL cholesterol/ total cholesterol ratio) was not significantly different. The hepatic and renal toxicity of MF was confirmed by serum AST, ALT, BUN, and CREA levels. Increased levels of AST, ALT, BUN, and CREA were found in the HFD group, but were decreased in the MF group, relative to the HFD group.

**Table 4:** Effects of *Melandrium firmum* Rohrback extract on obesity biomarkers in high‐fat diet‐fed mice

Groups^1^	NFD	HFD	GR	MF
Serum TG (mg/dl)	115.5 ± 11.6^2^ ^b^	120.9 ± 13.7^b^	120.3 ± 8.4^b^	84.55 ± 8.52^a^
Serum total cholesterol (mg/dl)	53.52 ± 4.23^a^	118.1 ± 5.67^b^	127.12 ± 8.30^b^	57.79 ± 3.05^a^
Serum HDL cholesterol (mg/dl)	43.17 ± 2.79^a^	78.81 ± 3.16^b^	79.45 ± 2.38^b^	40.04 ± 1.55^a^
HTR^3^	0.81 ± 0.02 ^c^	0.70 ± 0.01^b^	0.66 ± 0.02^a^	0.7 ± 0.01^b^
AST (U/l)	66.54 ± 15.29^a,b^	79.61 ± 15.78^b,c^	113.69 ± 15.75^c^	37.58 ± 1.74^a^
ALT (U/l)	31.85 ± 7.75^a^	89.52 ± 22.90^b^	70.43 ± 11.76^a,b^	23.77 ± 3.32^a^
BUN (mg/dl)	22.55 ± 0.78^c^	17.49 ± 0.32^b^	17.73 ± 0.47^b^	8.58 ± 0.21^a^
CREA (mg/dl)	0.41 ± 0.02^b^	0.45 ± 0.01^c^	0.42 ± 0.01^b^	0.21 ± 0.007^a^

^1^ NFD, normal‐fat diet; HFD, high‐fat diet; GR, HFD + 1% *Garcinia cambogia* extract; MF, HFD + 1% *Melandrium firmum* Rohrback.

^2^ Results are presented as means ± SE (*n* = 6); values indicated with different letters are significantly different from each other at *p* < .05.

^3^ HTR = HDL cholesterol/ total cholesterol.

### 3.5 Changes in weight and morphology of adipose tissue

**Figure 3:**
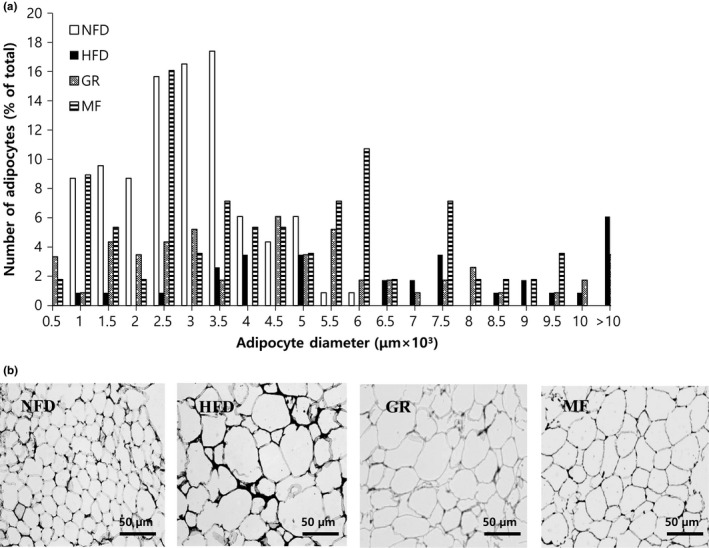
Effects of *Melandrium firmum* Rohrback extract on epididymal white adipose tissue (a) diameter and (b) microphology in high‐fat diet‐fed mice. Each specimen from the mice groups was fixed with 4% paraformaldehyde and sectioned into 4 μm, stained with H & E, and then viewed using light microscopy (magnification 200×). The image is a representative one. NFD, normal‐fat diet; HFD, high‐fat diet; GR, HFD + 1% *Garcinia cambogia* extract; MF, HFD + 1% *Melandrium firmum* Rohrback

The adipocyte size and adipose tissue weight were measured to investigate whether the weight‐reducing effect of MF was due to a decrease in fat mass. Significantly increased weight of the epididymal white adipose tissue was detected in the HFD group, when compared with the NFD group (Table 3), and the adipocyte size was larger in the HFD group than in the NFD group (Figure 3).

### 3.6 Effect of MF on the expression of genes related to adipogenesis and lipogenesis in white adipose tissue

**Figure 4:**
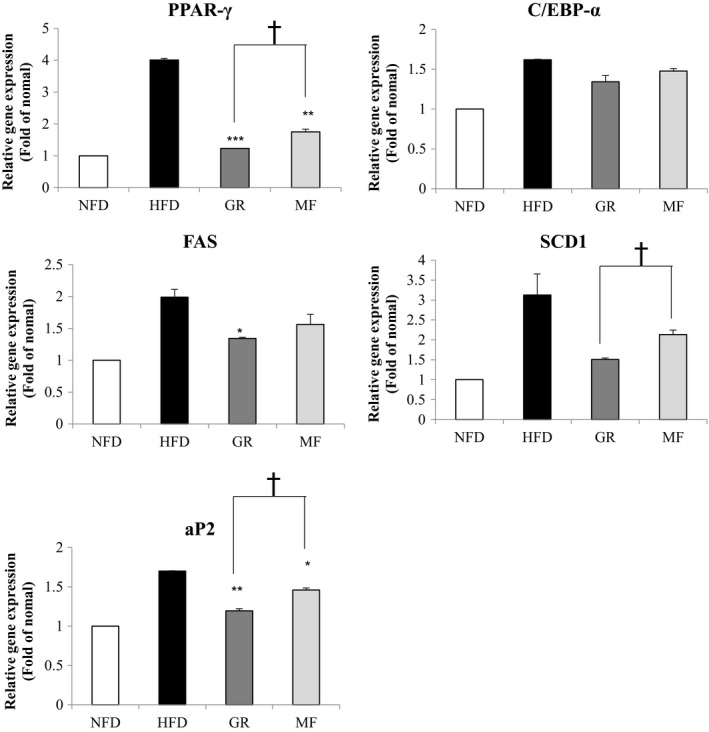
Effects of *Melandrium firmum* Rohrback extract on mRNA levels of adipogenic genes in epididymal white adipose tissue. Results are presented as mean ± SE. An asterisk indicates a significant difference, when compared with the HFD group (*, *p* < .05; **, *p* < .01; ***, *p* < .001). A dagger indicates significant difference between the GR and MF groups (†, *p* < .05). NFD, normal‐fat diet; HFD, high‐fat diet; GR, HFD + 1% *Garcinia cambogia* extract; MF, HFD + 1% *Melandrium firmum* Rohrback

To evaluate MF‐mediated reduction in adipocyte size, mRNA levels of genes related to adipogenesis and lipogenesis were measured. As shown in Figure 4, MF administration significantly down‐regulated the expression of PPAR‐γ and fatty acid‐binding protein 4 (aP2), which are involved in adipogenesis. The expression of lipogenic genes such as FAS and SCD‐1 was also reduced by MF administration, but the difference was not significant.

### 3.7 Change in liver weight and hepatic lipid metabolites

We examined the effect of MF on the change in liver weight and hepatic lipid metabolites in HFD‐fed mice because obesity may be one of the causes of development of a fatty liver. The liver weight in the HFD group was higher than in the NFD group, and MF administration decreased it by 1.26‐fold (Table 3). Hepatic lipid metabolites (fatty acids, phospholipid, lipid moieties, and cholesterol) were analyzed, and their levels were found to be increased in the HFD group (Table 5). MF administration decreased the levels of hepatic lipid metabolites, when compared with the HFD group. These results suggest that MF administration may inhibit lipid accumulation in the hepatic tissue.

**Table 5:** 1H NMR chemical shifts of endogenous lipid‐soluble hepatic metabolites

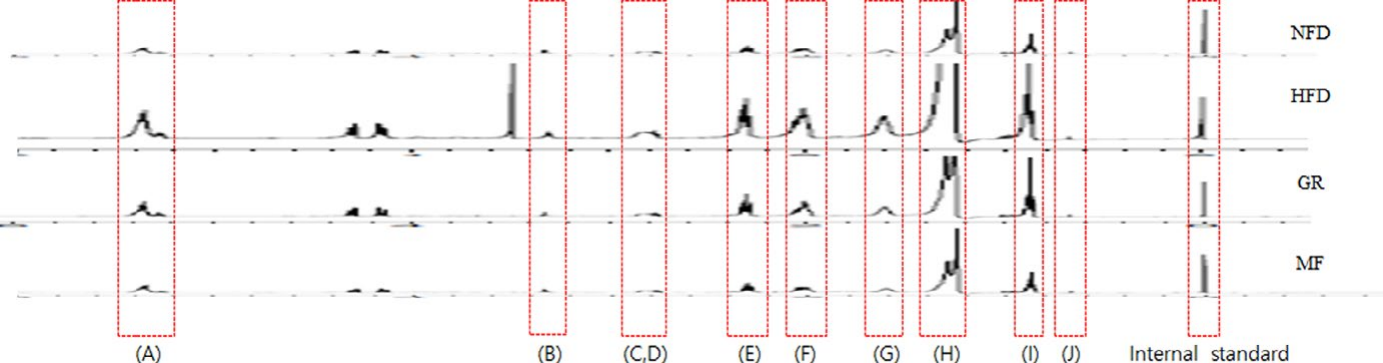							
	δ^1^H(ppm)	Type	Metabolites	Group^1^			
				NFD	HFD	GR	MF
(A)	5.32	Fatty acids	MUFA/PUFA	7.78 ± 0.80^2^	29.8 ± 4.38	9.63 ± 1.76	5.18 ± 0.33
(B)	3.16	Phospholipid	*N* ^+^(CH_3_)_3_	0.18 ± 0.004	2.11 ± 0.34	0.33 ± 0.003	0.32 ± 0.06
(C)	2.78	Lipid moieties	−CH = CH(−CH_2_CH = CH−)	3.56 ± 0.29	18.2 ± 4.98	3.96 ± 0.14	2.69 ± 0.18
(D)	2.72		−CH = CH−CH_2_CH = CH−				
(E)	2.27		αRCH_2_CH_2_CO	7.4 ± 1.02	31.67 ± 9.92	10.61 ± 2.46	5.28 ± 1.06
(F)	2		−CH_2_CH = CH−	6.92 ± 0.43	39.22 ± 11.61	12.91 ± 3.26	6.6 ± 1.5
(G)	1.55		βRCH_2_CH_2_CO	6.49 ± 0.2	23.61 ± 2.53	10.17 ± 2.48	5.08 ± 1.03
(H)	1.24		−(CH_2_)*_n_*−	54.65 ± 3.47	201.94 ± 32.30	78.24 ± 21.31	38.89 ± 9.16
(I)	0.83		RCH_3_	4.6 ± 0.55	33.57 ± 7.76	10.63 ± 2.27	5.58 ± 1.07
(J)	0.64	Cholesterol	Chol‐C18	0.05 ± 0.03	0.51 ± 0.35	0.20 ± 0.73	0.1 ± 0.03

^1^ NFD, normal‐fat diet; HFD, high‐fat diet; GR, HFD + 1% *Garcinia *cambogia extract; MF, HFD + 1% *Melandrium firmum* Rohrback.

^2^ Results are presented as mean ± SE (*n* = 6).

### 3.8 Effect of MF on the expression of genes related to lipogenesis in the liver

**Figure 5:**
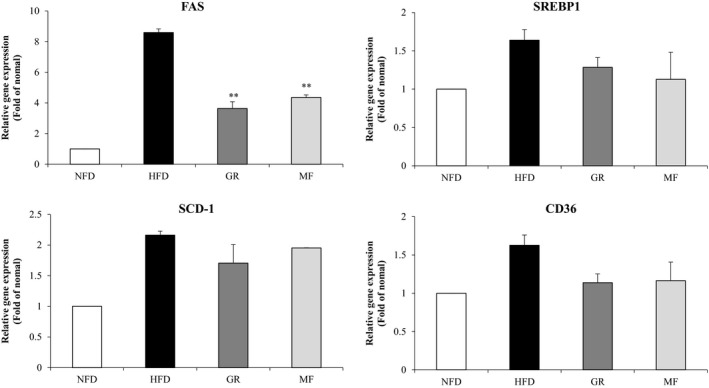
Effects of *Melandrium firmum* Rohrback extract on the mRNA levels of lipogenesis‐related genes in the liver. Results are presented as mean ± SE. An asterisk indicates a significant difference (**, *p* < .01). NFD, normal‐fat diet; HFD, high‐fat diet; GR, HFD + 1% *Garcinia cambogia* extract; MF, HFD + 1% *Melandrium firmum* Rohrback

To examine the effect of MF administration on the decline of hepatic lipid accumulation, we measured the mRNA levels of genes related to lipogenesis. MF administration significantly decreased the expression of lipogenesis‐related gene FAS, and the other genes that showed a decrease in the mRNA levels relative to the HFD group were SREBP‐1c, SCD‐1, and fatty acid translocase (CD36) in the hepatic tissue (Figure 5).

## 4 DISCUSSION

Obesity is a costly‐to‐treat condition and may be associated with negative social impression (Kwon et al., 2003); it may be associated with other metabolic disorders including type 2 diabetes and cardiovascular diseases (Kang et al., 2013). The ideal anti‐obesity drug is the once that leads to a sustained weight loss with minimal side effects (Rodgers et al., 2012). The cellular development of obesity‐associated adipose tissue involves both hyperplasia and hypertrophy (Ross & Desai, 2013). Hence, dietary supplements are used to regulate adipogenesis and lipogenesis to prevent obesity. In this study, to evaluate the potential effects of MF extract as a food supplement to prevent obesity, we focused on the regulation of adipogenesis and lipogenesis in vitro and in vivo.

A number of plant‐extract studies have shown anti‐obesity effects, and hence, we evaluated the anti‐adipogenic activity of MF in 3T3‐L1 cells; MF showed an anti‐adipogenic effect at concentrations of 10, 20, and 50 μg/ml. MF showed the strongest anti‐adipogenic effect at a concentration of 50 μg/ml. Additionally, there was a decrease in cell viability at this concentration, but this effect was not significant. Thus, MF was used to study the anti‐obesity effect in mice turned obese experimentally.

Body weight and fat reduction are important measures in an anti‐obesity experiment. HFD increased body weight, besides increasing TC, TG, AST, ALT, and reduced HTR in mice serum. MF decreased body weight and food intake significantly, but increased FER, when compared with the HFD group. The decreased food intake appeared in MF, and this result showed the possibility of anti‐obesity effect by loss of appetite. Du et al. (2010) also showed similar tendencies in the study because of loss of appetite by samples. However, in case of FER, MF showed a higher value compared to HFD, and we checked that other indicators related to obesity showed the tendency of decrease. Therefore, although the decrease of food intake due to the loss of appetite was showed, it seems that there is an anti‐obesity effect of MF itself.

These results indicated that MF has anti‐obesity effects. In addition, MF also reduced serum TG, TC, ALT, AST, BUN, and CREA levels. This effect of MF on body weight was induced by inhibition of fat accumulation without hepatic and renal toxicity.

Wang et al. (2017) showed that obesity is characterized by a simultaneous increase in fat cell number and size. MF treatment group showed comparable white adipose tissue weight, but adipocyte size decreased in comparison with the HFD group. Many genes are correlated with adipogenesis and lipogenesis. The major transcriptional genes PPAR‐γ, C/EBP‐α, aP2, FAS, and SCD‐1 play an important role in obesity (Wong, Kaneda, & Morita, 2014).

A number of reports have indicated that PPAR‐γ, C/EBP‐α, and aP2 are the major genes related to adipogenesis, while FAS and SCD‐1 are related to lipogenesis. Among them, PPAR‐γ is a central regulator of fat cell differentiation, and this gene is linked aP2 as adipose‐specific enhancer. Moreover, PPAR‐γ interacts with another transcription factor, C/EBP‐α, that is induced in adipogenesis and itself shows an adipogenic action (Spiegelman, 1998). FAS and SCD‐1 are induced by these transcription factors (Wang, Hudak, & Sul, 2010), and they are the key enzymes involved in fatty acid metabolism responsible for synthesizing palmitate (C16:0) (Wang et al., 2017). We performed RT‐PCR to analyze the change in adipogenesis and lipogenesis due to MF extract. In this experiment, MF reduced the expression of these genes PPAR‐γ, C/EBP‐α, aP2, FAS, and SCD‐1. These results suggest that MF administration suppressed adipogenesis and lipogenesis by decreasing these transcription factors in the adipose tissue.

**Figure 6:**
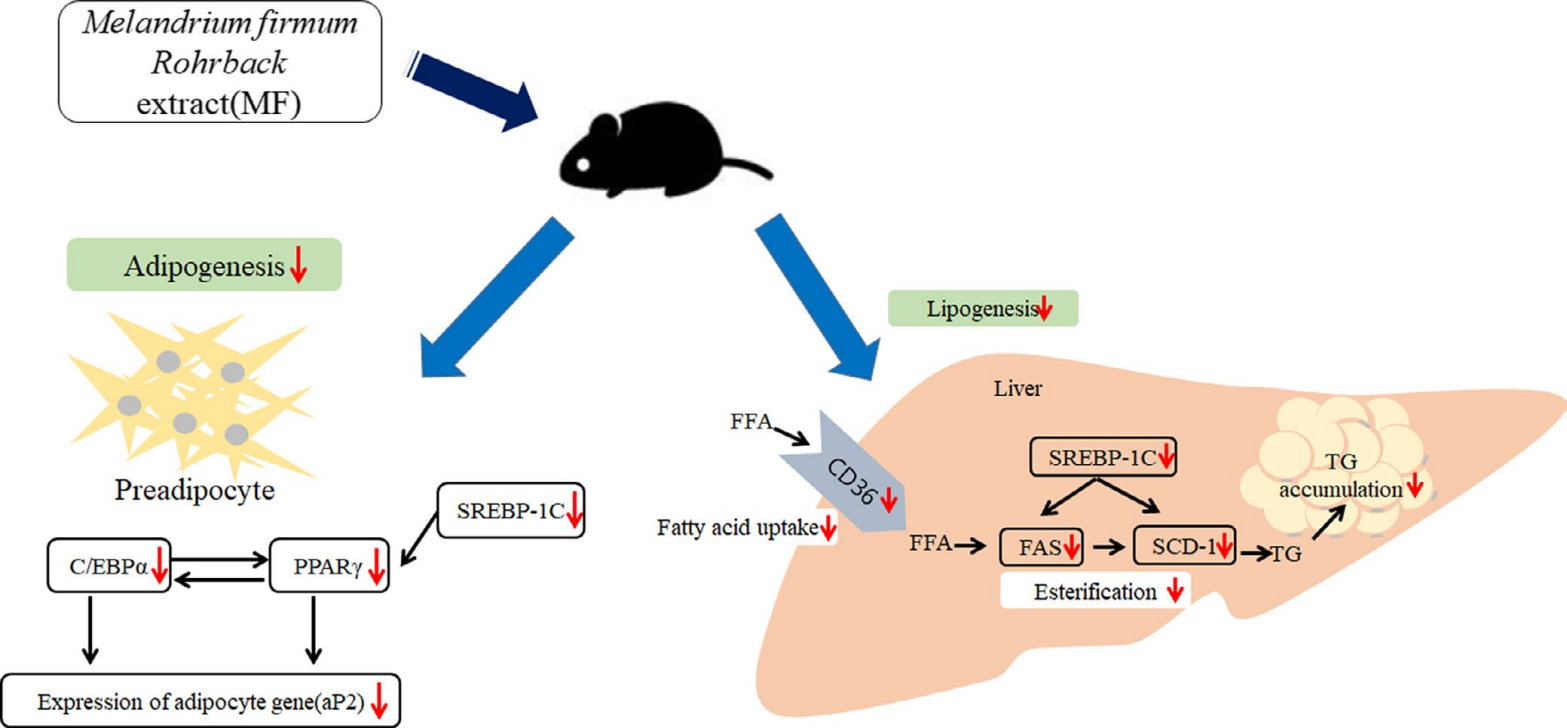
Mechanism of regulation of *Melandrium firmum* Rohrback extract on mRNA expression of adipogenic genes in white adipose tissue and liver

The liver fat content reflects lipogenesis (Adiels et al., 2006), and hepatic tissue weight is an important target for the anti‐obesity effect. The MF‐treated group showed decreased liver weight, when compared with the HFD group. The major parameters (SREBP‐1c, FAS, and SCD‐1 in the liver) related to obesity showed a decrease in the MF group. In addition, fatty acids, phospholipids, lipid moieties, and cholesterol levels were decreased in the liver tissue extract of the MF group, when compared with the HFD group. The increase in SREBP‐1c enhances fatty acid synthesis and accelerates triglyceride accumulation (Ahmed & Byrne, 2007). SCD‐1 induces obesity by catalyzing the desaturation of SFA palmitate and stearate to MUFA palmitoleate and oleate (Li, Berk, McIntyre, & Feldstein, 2009), and FAS and CD36 also play a regulatory role in lipid metabolism (Jung, Cho, Ahn, Jeon, & Ha, 2013). As shown in Figure 6, administration of MF shows an anti‐obesity effect by suppressing adipogenesis and suggests that it can be used to prevent hepatic lipid accumulation through the regulation of lipogenesis.

## CONFLICT OF INTEREST

The authors have no conflicts of interest to declare.

## ETHICAL APPROVAL

This study was approved by the Institutional Review Board of Hallym University.
